# Can natural detergent properties of bile acids be used beneficially in tackling coronavirus disease-19?

**DOI:** 10.2217/fvl-2020-0210

**Published:** 2020-12-15

**Authors:** Yashwant Kumar, Reena Yadav, Alka Bhatia

**Affiliations:** 1 ^1^Department of Experimental Medicine & Biotechnology, Postgraduate Institute of Medical Education & Research, Chandigarh, India; 2 ^2^Department of Immunopathology, Postgraduate Institute of Medical Education & Research, Chandigarh, India

**Keywords:** aerodynamics, antiviral, bile acids, chenodeoxycholic acid, coronavirus, detergent, envelope, respiratory, SARS-CoV-2, ursodeoxycholic acid

## Sometimes complex situations seek simple solutions

The coronavirus disease-19 is a pandemic caused by a beta coronavirus called SARS-coronavirus-2 (SARS-CoV-2). The virus usually affects the respiratory tract, causing an illness that may range from mild affection to severe acute respiratory symptoms leading to death. The virus is believed to enter the cells by binding to ACE2R and the damage caused by the virus has variously been attributed to its direct cytopathic effects as well as immune hyperresponsiveness (cytokine storm) to it. Though various drugs ranging from antiviral remdesivir to antimalarial hydroxychloroquine and convalescent plasma therapy have been proposed to be effective in reducing the viral load, till now, there is no effective therapy available.

SARS-CoV-2 is an enveloped, positive-sense, ssRNA virus. The viral structure like other beta coronaviruses comprises four proteins: nucleocapsid protein, membrane protein (M), an envelope protein (E) and spike protein. Out of the various proteins composing the structure of SARS-CoV-2, its S-glycoprotein has been most extensively studied as is believed to bind to the ACE2R and help in the ingress of the virus into the cell. Though E and M proteins are also believed to be important for viral entry they have not been very well characterized. Yet, a recent study has reported their similarity and differences from similar proteins of viral isolates from bats and pangolins [1,2].

The M and E proteins together form an envelope of the virus whereas the lipids are generally contributed by cellular membranes during the exit of the virus from cells. The lipid layer in enveloped viruses is believed not only to protect its genome but also, help in its invasion into the cell. Also, these viruses are believed to be more sensitive to environmental stressors like high temperature (>70°C), extremes of pH, etc. Their nucleic acid, proteins and lipids are supposed to be held together by non-covalent interactions that are broken by soap or detergent thus destroying the virus. Whereas the above is considered suitable for sanitization of hands and clothes, 1% sodium hypochlorite and 70% ethyl alcohol are usually recommended for the disinfection of various surfaces like plastic, cardboard, steel, wood, etc. Unfortunately, none of the above agents can be used in *in vivo* situations as they are all toxic chemicals. However, this does not prevent one from thinking, is there any naturally occurring endogenous disinfectant/detergent which can help in dealing with the virus with minimal or no toxicity [3,4]?

Bile acids and salts may be considered as natural detergents as they emulsify fats and modify the permeabilization properties of lipid membranes. While high concentrations of bile acids can cause lysis of the cell membrane, at lower doses, they have been found to facilitate delivery of drugs (Amphotericin B and Resveratrol) into the cells [5,6]. Due to their potential pharmaceutical applications, extensive efforts are being directed into synthesis of bile acids and their derivatives with improved carrier properties. These include the use of variety of chemical and enzymatic methods or the combination of above two to synthesize the pharmaceutically active ingredients like ursodeoxycholic acid. The latter was initially obtained from bile of black bear; however, subsequently, it was synthesized by chemical transformation in industries by using ketolithocholic acid as the precursor. The chemical method initially used involved reduction in *n*-propyl alcohol and metallic sodium; however, several modifications in the protocol have been done since then to increase the yield of ursodeoxycholic acid. Nowadays, cholic acid obtained from bovine source is used as a precursor for its synthesis [7].

Currently, bile acids are being used parenterally, orally as well as inhalationally for various cholestatic and other diseases. In the latter route, the bile salts have been found to affect the porosity of nanoformulations leading to aerodynamic properties which facilitate drug delivery into the lungs [8,9].

Bile acids are steroids synthesized from cholesterol in the hepatocytes and after conjugation with glycine and taurine are secreted in bile as bile salts. Conjugation makes them amphiphilic with both hydrophobic and hydrophilic properties. They later undergo enterohepatic circulation and help in the absorption of fat through micelle formation. Cholic acid and chenodeoxycholic acid are known as the primary bile acids whereas deoxycholic acid and lithocholic acid are the secondary bile acids formed by the action of bacteria in the intestine. Ursodeoxycholic acid, an epimer of chenodeoxycholic acid, is synthesized from it by dehydroxylation. It is considered as the most hydrophilic type of bile acid. Besides fat emulsification, bile salts have also been demonstrated to sub serve different metabolic functions in the body owing to the activation of different signaling pathways [10]. The following attributes may make bile acids suitable for SARS-CoV-2 targeting:

- First, the bile acids have been proposed to possess anti-inflammatory properties that may prove beneficial in curbing the cytokine storm believed to be involved in the pathogenicity of the virus. Immune modulation is believed to occur via involvement of Farnesoid X or the G-coupled receptor TGR5 at the outer plasma membrane. A study in cholestatic mice showed that excess bile acids in serum reduced the recruitment of monocytes and NK cells to the liver [11]. Another study revealed that bile acids, when modified by intestinal bacteria, may help in regulating the Treg numbers by altering the relevant immune signaling pathways [12];

- Second, bile acids have membrane permeability altering properties due to which they are extensively researched as drug carriers. Due to their amphiphilic nature, they are believed to act as ideal drug transporters. In the context of SARS-CoV-2, they may serve to increase the intracellular delivery and concentration of drugs like remdesivir or other antivirals when chemically conjugated to them [13]. Studies have shown that bile acids can be used intranasally though in low concentration (<0.3%) to facilitate absorption of other drugs, however, higher concentrations are believed to cause nasal irritation [14]. A study in rabbits showed that sodium tauroglycocholate increases the extent of nasal absorption of the antiviral drug acyclovir from 10% in the controls to 14.5%. This highlights their potential to be used in various inhalational formulations to fight the respiratory viruses [15];

- Third, it is known that the entry of viruses into the cell is blocked by drugs inhibiting viral envelope proteins or viral and entry receptor protein–protein interactions or protein–lipid interactions. Previous studies have shown that bile acids can incorporate themselves in between the membrane lipids thus altering their distribution and also the function of proteins attached to them [16]. In our preliminary work on chenodeoxycholic and ursodeoxycholic acid, the above bile acids were found to bind with receptor binding domain of S-glycoprotein of SARS-CoV-2 with negative binding energies (data not shown). This shows that bile acids have the potential to bind to SARS-CoV-2; however, the impact of their binding on the virus and its infectivity (ability to enter the cells) remains to be investigated;

- Fourth, some authors have proposed that bile acids may have virus replication modulating properties particularly in the context of hepatotropic viruses owing to activation of different signaling pathways and transcription factors [17]. Whether or not the bile acids can strip SARS-CoV-2 of its envelope by targeting its lipoprotein components, thus, destroying it completely remains interesting (though wishful thinking as of now) arena to explore.

A possible, although weak clue in favor of their protective role in SARS-CoV-2 infection may be that in the intestine the virus is less active due to the presence of bile acids. Therefore, though the gastrointestinal symptoms may be present they may appear late and are rarely life threatening. Also, there is no clear evidence for the affection of the liver or gall bladder by the virus despite the presence of ACE2R in these organs [18].

Literature search on the role of bile acids/salts specifically in the context of the respiratory system reveals studies mostly on manifestations after aspiration of bile acids and associated toxicity in cholestatic conditions or otherwise. Various toxic manifestations cited in these studies range from the increased propensity of respiratory infections to airway inflammation and pulmonary fibrosis [19–21]. A study by Aldhahrani *et al.* on human bronchial epithelial cell line (BEAS-2) showed that different bile acids in a concentration of 10–30 μm/l resulted in cell death and increased release of IL-6 and IL-8 [22]. However, in the above studies, the aspirated material was composed of a mixture of bile acids, cholesterol, gastric acid, etc., rather than bile acids alone. Further, a more recent study on inhalable albendazole conjugated with bile salts was not found to disturb lung surfactant function and aerodynamics in any way. Also, a study on house dust mite-induced mice models of allergic airway disease and human subjects with asthma demonstrated that conjugated bile salts via nasopharyngeal route have the potential to decrease airway inflammation [23]. Ursodeoxycholic acid is considered to be the least toxic bile acid due to its hydrophilic nature [24]. Perhaps, the dosage and type of bile acid (more hydrophobic or hydrophilic) needs to be standardized to least irritant and toxic levels before contemplating them as either standalone or combinatorial inhalational therapeutics in case of coronavirus disease-19.

Thus, whether naturally acting detergents like bile acids/salts can help in stripping off the envelope of SARS-CoV-2 thereby disrupting its assembly is a million-dollar question. Although a review of past literature and logical thinking throws light on their potential benefit, only time and an experimental proof will be able to provide definitive answers to the above. Future studies may be planned with special emphasis not only on investigating their probable benefit but also on understanding issues like exact mechanism of action and administration strategies etc. However, we believe that it is worthwhile to investigate them as one of the many potential inhalation based preventive or therapeutic candidates for further research and study.
